# Inferring the historical demography of southern African cheetahs (*Acinonyx jubatus*) using Bayesian analyses of molecular genetic data

**DOI:** 10.1590/1678-4685-GMB-2024-0253

**Published:** 2025-05-19

**Authors:** Ezequiel Chimbioputo Fabiano, Sandro Luis Bonatto, Anne Schmidt-Küntzel, Stephen J. O’Brien, Laurie Marker, Eduardo Eizirik

**Affiliations:** 1 Cheetah Conservation Fund, Otjiwarongo, Namibia.; 2 Pontifícia Universidade Católica do Rio Grande do Sul, Escola de Ciências da Saúde e da Vida, Laboratório de Biologia Genômica e Molecular, Porto Alegre, RS, Brazil.; 3 University of Namibia, Ngweze, Katima Mulilo, Namibia.; 4 Nova Southeastern University, Fort Lauderdale, USA.

**Keywords:** Coalescent modelling, Felidae, population decline, Namibia, microsatellites

## Abstract

The contemporary genetic diversity of the cheetah (*Acinonyx jubatus*) has been the focus of several studies, which have revealed very low levels of variation. Different hypotheses have been proposed to explain this pattern of low diversity, and require additional scrutiny. Here, we used published microsatellite data and coalescence-based analytical methods to explore the historical demography of the largest free-ranging cheetah population, aiming to assess whether present-day diversity may have been impacted by a historical demographic decline. Our results support the hypothesis of a historical (and most likely gradual) demographic decline over the past ~10,000 years, leading to a present-day *N*_*e*_ ranging from 700 to 1,600 individuals. This decline was likely induced by climate-driven vegetational shifts affecting habitat suitability and possibly also interspecies interactions with prey and competitors. These results help clarify the demographic history of cheetahs in southern Africa and its impact on the current genetic diversity of this population.

The historical demography of a species reflects the changes in its population size over time. It plays a crucial role in the survival of species, as reductions in population size negatively affect levels of genetic diversity, and low levels of genetic diversity are often linked to reduced fitness. Moreover, understanding past fluctuations, and their relationships with present-day genetic diversity, is relevant in the context of understanding present-day dynamics and predicting likely outcomes of future scenarios.

Reductions in population size are often caused by external factors such as climate change, habitat degradation, prey-predator dynamics, and disease outbreaks. Throughout the Quaternary period (2.5 million years ago to the present), the climate was highly heterogeneous in Africa, with that of western and eastern Africa being relatively unstable compared to southern Africa (Stokes et al., 1997; Maslin et al., 2012). This variation in climate was accompanied by changes in species-specific habitat suitability and has likely affected the contemporary genetic diversity of many species (Hewitt 2004; Chase et al., 2010). Comparative phylogeography across taxa indicates that southern Africa was a refugium from which populations recolonized more northerly regions (Lorenzen et al., 2010; Bertola et al., 2011; Schwab et al., 2011). But even in southern Africa, notable oscillations between wet and dry periods have been reported in the Holocene (Hewitt 2000). Since responses can be species- or population-specific (Lorenzen *et al.*, 2011; Kim et al., 2016; Nadachowska‐Brzyska et al., 2016), assessing comparative historical patterns should benefit from a growing body of studies targeting different taxa.

One species that seems to have a particularly interesting demographic history is the cheetah (*Acinonyx jubatus*), for which remarkably low levels of genetic diversity were first identified in the early 1980s (O’Brien et al., 1983, 1985) and more recently confirmed by studies employing genome-wide data (Dobrynin et al., 2015; Prost et al., 2022). While it is widely recognized that the origin of the cheetah’s low extant genetic diversity is predominantly the result of events predating modern civilization, different hypotheses have been proposed to account for this phenomenon. Early genetic studies using various molecular markers (allozymes, mtDNA restriction fragment length polymorphisms, minisatellite fingerprinting) and samples from southern and eastern Africa, hypothesized that the low diversity was likely a consequence of one or more bottlenecks at the end of the Pleistocene (12,000 - 10,000 years ago [ya]) (O’Brien *et al.*, 1983, 1987; Menotti-Raymond and O’Brien, 1993). Two alternative hypotheses to a reduction in population size as cause for the low diversity were subsequently proposed. First, that the low diversity could have been due to the persistence of the species at low effective population size (*N*_*e*_ ), induced by the high reproductive variance observed in species with a polygynous mating system (Pimm et al., 1989). Second, that it could be due to a continuous cycle of extinction of subpopulations followed by re-colonization of the areas, following a metapopulation dynamics (Pimm *et al.*, 1989; Gilpin, 1991; Hedrick, 1996). In these two alternative hypotheses, the low diversity could have been maintained over a long period without implying a detectable historical population reduction. Subsequent genetic studies using microsatellite and MHC loci were in agreement with the inference of an ancestral bottleneck (e.g., Driscoll et al., 2002; Castro-Prieto et al., 2011).

During the past decades, there has been a surge of advances in computational methods exploring the historical demography of modern populations using empirically collected molecular data. Of particular interest is the application of the coalescent and Bayesian approaches in population genetics (e.g., Luikart and England, 1999; Storz and Beaumont, 2002; Beaumont MA, 2010; Lopes and Beaumont, 2010). These methods have now been widely used to model changes in *N*_*e*_ of many different species, including the cheetah (Okello et al., 2008; Phillips et al., 2012; Quéméré et al., 2012; Dobrynin et al., 2015). Results based on the analyses of whole genome data support a historical decline in the cheetah population size as the cause for its low genetic diversity, rather than a persistently low effective population sizes or a metapopulation dynamics (as proposed by the alternative hypotheses mentioned above). At the same time, while population decline was supported, even with complete genomes the mode of decline could not be clearly determined (Dobrynin *et al.*, 2015). Different analytical approaches (Dadi *vs.* PSMC) supported a sharp or a gradual decline, respectively (Dobrynin *et al.*, 2015), indicating that this issue remains incompletely resolved.

To investigate the support for these hypotheses using an independent dataset, here we assessed the demographic history of the Namibian cheetah population using coalescent-based methods applied to a large, previously published microsatellite data set (Marker et al., 2008). Namibia has one of the largest remaining cheetah populations, estimated at 1,500 adult and adolescent individuals, and connected to the broader southern Africa contiguous adult population of ~3,500-6,800 individuals (Charruau et al., 2011; Durant et al., 2017, Weise et al., 2017). This population was considered appropriate for the study due to its relatively large census size and being panmictic (Marker *et al.*, 2008). Panmixia is crucial, as it reduces the risk of false signals of bottleneck caused by sub-structuring (Sousa et al., 2009; Peter et al., 2010), while larger current sizes reduce the likelihood of the population having experienced high genetic drift in the recent past (Tallmon et al., 2010).

Our data set comprises 89 unrelated individuals (determined based on behavioral data, parentage analyses and estimates of genetic relatedness) and originally contained 38 microsatellite loci (Marker et al., 2008). For this study, we only included the 29 loci that showed modal allelic distribution. We used this dataset to assess historical trends in population size using four coalescent-based approaches, implemented in the programs MSVAR 1.3 (Storz and Beaumont, 2002), LAMARC v2.1.2b (Kuhner, 2006), Migrate-n v.3.6.6 (Beerli and Palczewski, 2010) and VAREff v1.2 (Nikolic and Chevalet, 2014). These approaches employ a coalescent framework based on the Wright-Fisher model (Moyer et al., 2012; Sharma et al., 2013), an assumption which our study population approximates (Marker *et al.*, 2008).

We initially employed MSVAR1.3 to test whether this dataset supports any inference of changes in *N*_*e*_ in this population and, if so, whether this inference was robust to varying model parameters. We ran six independent analyses varying in chain length (2 × 10^9^ or 4 × 10^9^ steps) and spacing among sampled states (1 × 10^5^ or 2 × 10^5^, respectively), in every case yielding 10,000 samples after a 50% burn-in. We also varied the assumed mutation rate (10^-6^ or 10^-3^) (Li et al. 2002), the assumed demographic trend in the prior model (stable or expanding size), and in case of demographic change, whether it was linear or exponential.

Subsequently, we used LAMARC to estimate trends in this population’s long-term *N*_*e*_ and demographic exponential growth rates (*g*). Two independent runs, each of 2 × 10^9^ steps were performed, with 20,000 recorded parameters sampled every 10^5^ steps, with 10% additional steps employed as burn-in. Priors for the population diversity parameter ‘theta’ (θ) and *g* were drawn from uniform distributions, and ranged from 1 × 10^-5^ to 10 and -500 to 500, respectively. Convergence of the runs was assessed using the program Tracer v1.4 (Rambaut and Drummond, 2007). Long-term effective population size was determined by solving the formula θ = 4*N*_*e*_ µ, where θ is the estimated population diversity parameter, *N*_*e*_ is the effective population size and µ is the mean dinucleotide microsatellite mutation rate per locus per generation (5.6 × 10^-4^; Weber and Wong, 1993). Generation time was assumed to be 6 years (Marker and O’Brien, 1989).

We then used the Bayesian inference implemented in Migrate-n to estimate the present-day θ and the demographic history (via skyline plot) of this cheetah population. The main parameters used were: 8 million generations sampled every 200 steps, resulting in 40,000 recorded steps. Burn-in was 500,000 steps. The prior distribution of θ was set to uniform between 0 and 15.

Finally, we used VarEff to estimate past changes of effective population size using approximate likelihoods in a Markov Chain Monte Carlo approach. We employed the command ‘Theta’ with the following parameters: Theta (NBLOC = 30, JMAX = 5, MODEL = ‘G 0.15’, MUTAT = 0.00056, NBAR = 2000, VARP1 = 4, RHOCORN = 0, GBAR = 10000, VARP2 = 3, DMAXPLUS = 8, Diagonale = 0.5, NumberBatch = 10000, LengthBatch = 20, SpaceBatch = 20, Burnin = 50000, AccRate=0.25). Mutation rate and generation time were assumed as described above.

All MSVAR runs supported a scenario of population decline, irrespective of the assessed variations in model parameters and demographic assumptions (Figure 1). This included runs in which the population was assumed to have expanded in the prior model, and which still yielded an inference of demographic decline.

**Figure 1 -:**
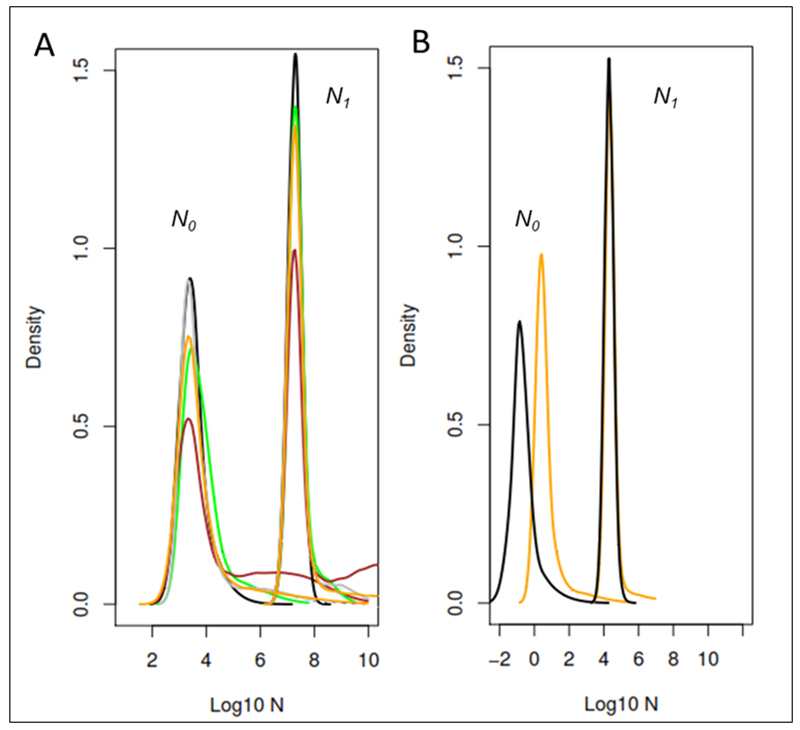
Posterior distribution of present (*N*_*0*_ ) and past (*N*_*1*_ ) effective population sizes, derived from independent runs using MSVAR1.3. (A) assumes a microsatellite mutation rate of 10e-6 and exponential changes; black and green lines represent runs that assumed the population to have expanded, while brown and gray to have remained stable; orange represents the combined runs. (B) Results from two independent MSVAR1.3 long chains (4 × 10^-9^ iterations each) in which the population was modeled as stable but allowed to change exponentially (orange) or linearly (black). *N*_*0*_ , *N*_*1*_ = recent, ancestral population sizes, respectively.

Consistently, the LAMARC results also supported a decline scenario by yielding a negative estimated growth rate of -0.2 (95% CI: -0.15 - -0.05). Although this rate of decline is small, its confidence interval did not overlap zero, implying that this approach strongly supported the inference that the population size was larger in the past. In addition, the LAMARC result yielded a low value for present-day θ (3.44 [95% CI: 3.13 - 3.72]), which translates to an estimated *N*_*e*_ of 1,537 (CI: 1.399 - 1.661) individuals. 

The Migrate-n skyline plot depicted a continuous declining trend, from an *N*_*e*_ of ~40,000 individuals *ca.* 16,000 ya to ~3,500 individuals *ca.* 150 ya (Figure 2). Although values older than *ca.* 6,000 ya are based on small sampling and thus less reliable, the overall trend of decline was robust throughout the whole period, slowing down *ca.* 1,500 ya. Migrate-n estimated the current θ of the Namibian cheetah population as 3.65 (95% CI = 3.29-4.67) which translates to an *N*_*e*_ of 1,629 (1,469-2,085), similar to the results estimated with LAMARC.

**Figure 2:**
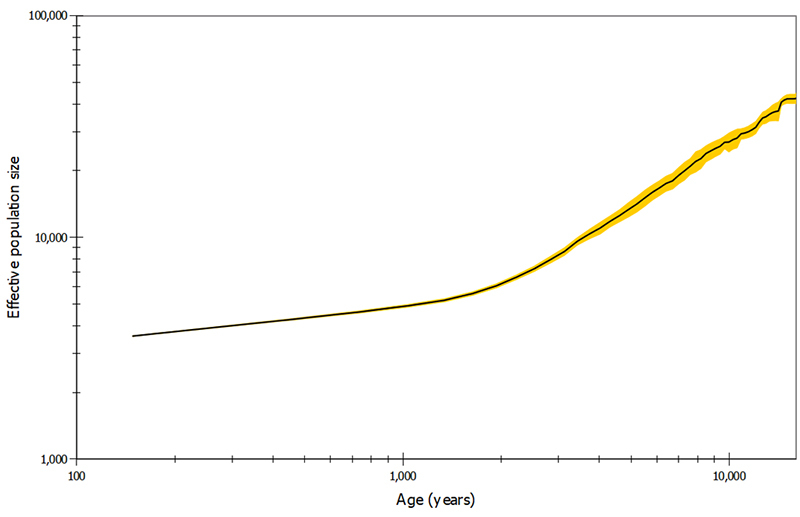
Skyline plot generated with Migrate-n, presenting estimated *N*_*e*_ values between 16,000 and 100 ya in log scale. The black line is the median estimate and the yellow band shows the 95% highest posterior density intervals.

The VarEff results also reconstructed a trend of reduction of this cheetah population’s *N*_*e*_ in the last 10,000 years (Figure 3), consistent with the Migrate-n and LAMARC results. This trend appears to have been continuous (using both the harmonic mean and median estimates). Prior to *ca*. 10,000 ya, VarEff showed a population increase that started *ca*. 30,000 ya, after which the decline ensued. Overall, VarEff suggested smaller population numbers, with a present-day *N*_*e*_ of the Namibian cheetah population estimated to be of only *ca*. 700-1000 individuals.

**Figure 3:**
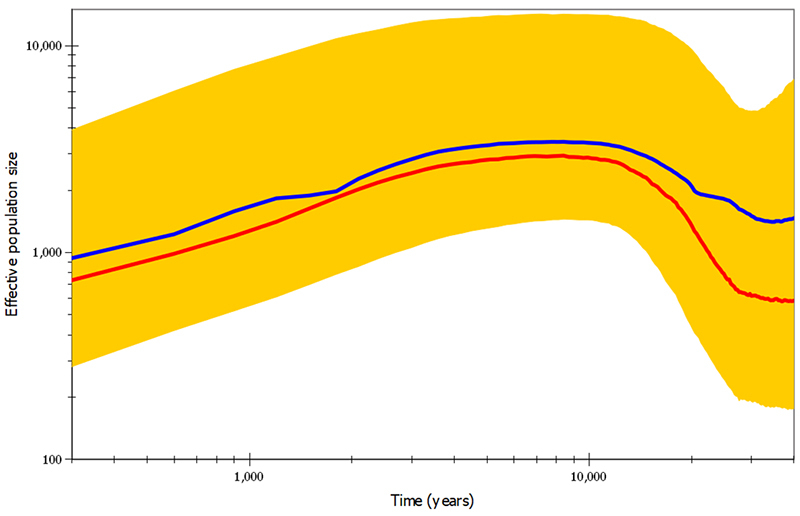
VarEff skyline plot presenting estimated *N*_*e*_ values between 40,000 and 100 ya in log scale. The red and blue lines are the harmonic mean and the median estimates, respectively, and the yellow band shows the 95% highest posterior density intervals.

Overall, our results were very consistent in the reconstruction of the demographic history of Namibian cheetahs, which represent the broader southern African population considering their connectivity across this region (Charruau et al., 2011). We initially focused on the question of whether an independent genetic dataset (relative to the genome analyses reported by Dobrynin et al. 2015) supports the hypothesis of a historical demographic decline in this species. While there was some variation among the estimates derived from our four methods (MSVAR, LAMARC, Migrate-n, VarEff), all analyses supported a historical decline in this cheetah population. Two of the methods (Migrate-n and VarEff) allowed a reconstruction of its demographic trajectory, with concordant indication of a decline beginning at least ~10,000 years ago (see Figures 2 and 3).

The magnitude and rate of decline varied among the analyses. While LAMARC estimated a mild but significant rate of decline (-0.2), Migrate-n and VarEff retrieved a 4 to 10-fold decrease in *N*_*e*_ over a 10,000-year period (i.e., ~30,000 to ~3,500; ~3,000 to ~700 individuals, respectively). Although the estimated absolute timing of those demographic changes would be affected by the assumed mutation rate, microsatellite mutation rates are species- and locus-specific (Ellegren, 2004), and no specific rates have been directly ascertained for cheetahs. While assuming different rates would change the timeframe of inferred shifts in population size, the conclusion regarding the declining trend would still hold.

Given the consistent support for a historical decline, our second focus was to assess whether this decline was gradual or abrupt. Our findings consistently support a gradual decline, in agreement with the results of a PSMC analysis using whole genome data (Dobrynin et al., 2015). We also observed a recent attenuation of the rate of population decrease in the Migrate-n skyline plot (Figure 1), suggesting that the process of demographic decline may have occurred at different paces over time.

Overall, our results indicate that the low levels of genetic diversity in present-day cheetahs (at least in southern African populations) can be explained by the inferred historical demographic decline. Accordingly, we observed no evidence for the long-term maintenance of *N*_*e*_ at low levels, as would be expected from the alternative hypotheses that imply either high reproductive variance due to the cheetah’s polygynous mating system, or a continuous metapopulation dynamics (Pimm et al., 1989; Gilpin, 1991; Hedrick, 1996).

An additional inference based on our analyses pertains to the estimates of present-day *N*_*e*_ in this cheetah population (which likely represents the genetically continuous southern African population; Weise et al., 2017). The estimates were consistently low across all methods, ranging from 700-1000 individuals for VarEff to 1,537 for LAMARC and 1,629 for Migrate-n. It is frequently accepted that *N*_*e*_ tends to be 5-10 times smaller than the census size of a population (Frankham et al, 2014), which would suggest that cheetah census numbers in the region (southern Africa) range from 3,500 to 16,000 individuals. These values are comparable with the overall estimated numbers for cheetahs in this area, whose latest estimate for adult individuals alone was 3,500-4,000 (Durant et al., 2017; Weise *et al.*, 2017). The congruence between this census estimate and our *N*_*e*_ -based estimate indicates robustness of the genetic assessments of present-day diversity and previous demographic trends leading up to this inferred scenario. In addition, this congruence highlights the usefulness of available genetic data, including those obtained with microsatellite markers, to estimate demographic parameters from present-day populations.

The inferred demographic decline of southern African cheetahs has likely been driven by one or more ecological processes, such as climate-induced vegetational shifts and changes in inter-species dynamics with prey and/or competitors (including humans). Climate-driven habitat shifts in this time frame have been pervasive in Africa (Stokes et al., 1997; Hewitt 2000; Hewitt 2004; Chase et al., 2010; Lorenzen et al., 2010; Bertola et al., 2011; Schwab et al., 2011; Maslin et al., 2012), and may have affected cheetah densities directly. Furthermore, it is plausible that interspecific competition with lions (*Panthera leo*), leopards (*Panthera pardus*), and spotted hyenas (*Crocuta crocuta*), the cheetah’s main competitors (Durant, 2000; Walker et al., 2022), were exacerbated during times of reduced habitat and prey availability. In addition to the historical declines detected in this study, cheetahs have undergone severe recent population declines, largely attributed to anthropogenic threats and habitat reduction (Marker-Kraus et al., 1996; Durant *et al.,* 2017). Overall, the results from this study provide useful information for improving our understanding of the cheetah’s long-term demography, especially for populations in southern Africa, and highlight the importance of genetic research on this complex and threatened species.
